# All-cause and cause-specific mortality in inflammatory bowel disease across the biologic era: a population-based matched cohort study

**DOI:** 10.1093/ecco-jcc/jjag105

**Published:** 2026-07-13

**Authors:** Onuma Sattayalertyanyong, Zoann Nugent, Charles N Bernstein

**Affiliations:** Department of Medicine, Division of Gastroenterology, Faculty of Medicine Siriraj Hospital, Mahidol University, Bangkok, Thailand; IBD Clinical and Research Centre, University of Manitoba, Winnipeg, MB, Canada; IBD Clinical and Research Centre, University of Manitoba, Winnipeg, MB, Canada; Department of Medicine, Max Rady College of Medicine, Rady Faculty of Health Sciences, University of Manitoba, Winnipeg, MB, Canada; IBD Clinical and Research Centre, University of Manitoba, Winnipeg, MB, Canada; Department of Medicine, Max Rady College of Medicine, Rady Faculty of Health Sciences, University of Manitoba, Winnipeg, MB, Canada

**Keywords:** inflammatory bowel disease, Crohn’s disease, ulcerative colitis, all-cause mortality, cause-specific mortality

## Abstract

**Background & Aims:**

Inflammatory bowel disease (IBD) is associated with increased mortality, but whether death rates and causes of death have changed across the biologic era is unclear. We aimed to assess all-cause and cause-specific mortality in Crohn’s disease (CD) and ulcerative colitis (UC) across therapeutic eras.

**Methods:**

We conducted a retrospective, population-based matched cohort study (1984-2019). IBD cases were matched 1:10 to unaffected controls by age, sex, and geography. Follow-up was stratified into a pre-biologic era (1984-2000) and biologic era (2001-2019). All-cause mortality was assessed using Cox models and cause-specific mortality using competing-risk methods.

**Results:**

We identified 13 306 patients with IBD (5955 CD; 7351 UC) and 133 060 matched controls. During follow-up, 2156 patients with IBD (16.2%) and 19 095 controls (14.4%) died. CD was associated with higher all-cause mortality than controls (hazard ratio [HR] 1.28, 95% confidence interval [CI] 1.19-1.37). UC mortality did not differ from controls (HR 1.03, 95% CI 0.97-1.10). In era-stratified analyses, mortality was comparable in the pre-biologic era (IBD HR 1.04, 95% CI 0.93-1.15). In the biologic era, excess mortality was observed in CD (HR 1.34, 95% CI 1.24-1.45) but not in UC (HR 1.05, 95% CI 0.98-1.12). Compared with controls, CD had higher mortality from colorectal cancer (HR 2.28), renal disease (HR 2.79), non-Hodgkin lymphoma (HR 1.89), and sepsis (HR 2.12), while UC had higher mortality from colorectal cancer (HR 1.56) and cholangiocarcinoma (HR 3.21).

**Conclusions:**

All-cause mortality was modestly higher in CD, while UC mortality was similar to matched controls. The CD mortality gap appeared most evident with longer follow-up.

## Graphical abstract

**Figure:**
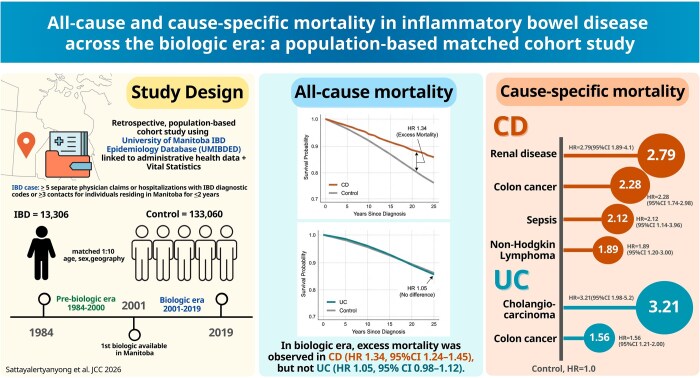

## 1. Introduction

Inflammatory bowel disease (IBD), including Crohn’s disease (CD) and ulcerative colitis (UC), is a chronic, immune-mediated disorder of the gastrointestinal tract.^1^^,^^2^ Over the past 2 decades, population-based studies and global burden analyses have documented a sustained rise in the incidence and prevalence of IBD across North America, Europe, and many newly industrialized countries.^3–9^ As the prevalence increases, the long-term burden of disease becomes increasingly apparent: cumulative intestinal injury contributes to surgery, recurrent hospitalizations, functional impairment, and premature death.^9–16^

Meta-analyses of population-based cohorts generally demonstrate a modest increase in all-cause mortality in IBD, with excess risk driven predominantly by CD (standardized mortality ratios [SMR] ∼1.3-1.5), whereas mortality in UC is typically not different from that of the general population.^13^^,^^17–19^ Subsequent population-based studies from North America, Europe, and Asia have further supported increased mortality in IBD, with elevated deaths attributed to digestive diseases, malignancy, respiratory disease, and infections, while associations with cardiovascular mortality have been inconsistent across settings.^15^^,^^16^^,^^20–33^ Several clinical and demographic factors have been linked to higher mortality risk, including prior gastrointestinal surgery, concomitant primary sclerosing cholangitis, very early onset disease (diagnosis before 6 years of age), male sex, older age, underweight, and corticosteroid exposure.^16^^,^^24^^,^^26^^,^^27^^,^^31^^,^^34–36^ In contrast, 5-aminosalicylic acid and biologic therapy have been reported as protective in 1 analysis.^34^

Therapeutic innovation in IBD has aimed to improve long-term outcomes by sustaining remission, reducing surgery and hospitalization, and ultimately lowering mortality. A meta-analysis by Canavan et al. reported an SMR of 2.2 in CD, with a 2.7% per-year decrease (95% confidence interval [CI] 1%-4%, *P* = .003) among persons diagnosed before 1970, coinciding with the introduction of corticosteroids and, later, immunomodulators. However, among persons diagnosed after 1970, the SMR remained stable at ∼1.4-1.5 without a significant decline during follow-up into the 2000s.^19^ Although corticosteroids are effective for induction therapy and may reduce short-term surgical risk, prolonged exposure is associated with increased mortality.^36^^,^^37^ Biologics were introduced for IBD management in the United States in 1998 and were adopted internationally through the 2000s.^9^ Compared with conventional therapy, biologics provide higher efficacy for induction and maintenance of remission.^38^^,^^39^ Although serious infections were reported in nearly half of patients treated with biologics, this was not accompanied by increased mortality; indeed, mortality may have been lower than with prolonged corticosteroid therapy.^36^^,^^40^

Despite these advances, the impact of modern IBD therapy on long-term survival remains uncertain. Adult-onset and elderly-onset cohorts from Sweden suggest that although relative mortality risk has decreased over the past 5 decades—particularly early after diagnosis—patients treated in the ­biologic era continue to experience excess mortality compared with the general population.^25^ Moreover, few studies have explicitly compared IBD mortality with matched controls across calendar periods within the same population and healthcare system. Cause-specific mortality patterns, including colorectal cancer, hepatobiliary malignancy, cardiovascular disease, sepsis, and renal disease, also remain incompletely characterized across earlier and later treatment periods. To address these gaps, we conducted a retrospective, population-based matched cohort study to compare all-cause mortality in IBD versus matched controls across pre-biologic and biologic periods and to examine differences in cause-specific mortality between these calendar eras.

## 2. Methods

### 2.1. Study design and population

This retrospective, population-based cohort study used the University of Manitoba IBD Epidemiology Database (UMIBDED) linked to provincial administrative health databases, including the Manitoba Health administrative databases, the Drug Program Information Network (DPIN), and the Manitoba Vital Statistics database. UMIBDED identifies all residents of Manitoba with IBD using a unique 9-digit personal health identification number (PHIN), which enables linkage across health system encounters captured from April 1, 1984 onward. Manitoba Health provides universal health insurance coverage for all provincial residents.

IBD cases were identified from Manitoba Health administrative data using a validated case definition requiring  ≥5 separate physician claims or hospitalizations with IBD diagnostic codes (or  ≥3 contacts for individuals residing in Manitoba for ≤2 years), with previously reported sensitivity and specificity of approximately 90%. IBD subtypes were classified as CD (ICD-9-CM 555.xx, ICD-10 K50) and UC (ICD-9-CM 556.xx, ICD-10 K51).^41^ Controls were selected from Manitoba residents in the same provincial health insurance system who had no recorded IBD diagnosis and were matched to cases on age, sex, and geographic region at an approximate 10:1 ratio. The final study cohort included 13 306 individuals with IBD (5955 CD; 7351 UC) and 133 060 matched controls.

Death and cause of death were identified through linkage of UMIBDED to the Manitoba Vital Statistics Registry. Date of death was available through November 30, 2024, whereas underlying cause of death was available through September 30, 2023. Cause-specific analyses were therefore restricted to deaths occurring up to September 30, 2023.

We included all eligible cases during 1984-2019 and evaluated outcomes across 2 calendar periods: the pre-biologic era (1984-2000) and the biologic era (2001-2019). The year 2001 was selected as the start of the biologic era because biologic therapies for IBD were first recorded in the Manitoba drug database in 2001. The study was approved by the University of Manitoba Research Ethics Board and the Health Information Privacy Committee of Manitoba Health.

### 2.2. Outcomes and analysis

The primary outcome was all-cause mortality in IBD compared with matched controls, with analyses stratified by calendar era (biologic-era care defined as 2001 onward). Secondary outcomes were underlying cause-specific mortality and assessment of era-related differences in cause-specific mortality patterns.

Time-to-event analyses used time since IBD diagnosis as the time scale. For controls, follow-up began on the index date corresponding to the matched case’s IBD diagnosis date. Individuals were followed until death, out-migration from Manitoba, or end of study (December 31, 2019), whichever occurred first. For descriptive comparisons, crude mortality rates were calculated as the number of deaths per 100 000 person-years. To provide age-adjusted absolute mortality estimates, age-standardized all-cause mortality rates were also calculated per 1000 person-years and standardized to the 2001 Canadian Census population. Rates were summarized in multi-year calendar intervals to improve stability, as annual estimates were unstable because of small event counts.

Era-specific analyses were conducted in 2 complementary ways. First, crude mortality rates were summarized for cases and controls within each era. Second, in time-to-event analyses, the pre-biologic era analysis censored follow-up at December 31, 2000, whereas the biologic era analysis included follow-up from January 1, 2001 onward. To address potential era mixing, we performed diagnosis-era sensitivity analyses by stratifying patients according to diagnosis period and follow-up window: diagnosis before 2001 with follow-up censored at 2000, diagnosis after 2001 with follow-up during the biologic era, and diagnosis before 2001 with long-term follow-up after 2000. Sex-stratified Kaplan–Meier curves compared survival between cases and matched controls by sex and IBD subtype; for these plots, individuals diagnosed before 1987 (and their matched controls) were excluded to ensure complete capture of diagnostic history.

We also performed descriptive sensitivity analyses of biologic exposure during the biologic era, comparing mortality patterns among IBD cases and matched controls according to biologic exposure status.

All-cause mortality comparisons were evaluated using Cox proportional hazards models, with results reported as hazard ratios (HRs) and 95% CIs. Cause-specific mortality was evaluated using competing-risk methods, in which deaths from all other causes were treated as competing events for each specific cause of interest. Multivariable models included age and sex, and evaluated associations with IBD subtype (CD vs. UC) and era (biologic vs. pre-biologic). For analyses in the control population, all-cause mortality models were fitted using the full matched control cohort. Cause-specific competing-risk models were fitted using a 10% random sample of controls for computational efficiency, with identical model specification and covariates as those used in the IBD cohort. Statistical significance was defined as a 2-sided *P* < .05. The analysis was performed using SAS Institute V.9.4 (Cary, North Carolina, USA).

## 3. Results

### 3.1. Baseline characteristics

The cohort included 13 306 patients with IBD and 133 060 matched controls. During follow-up, 2156 persons with IBD (16.2%) and 19 095 controls (14.4%) died. Persons with CD were diagnosed at a younger age (median 34 [23-49] years) than those with UC (median 40 [28-56] years). Age at death was lower among persons with CD than their matched controls (median 72 [60-82] vs. 75 [63-84] years; *P* < .0001), whereas age at death was similar between persons with UC and controls (median 77 [65-86] vs. 77 [65-85] years; *P* = .54). When stratified by era, age at death in CD was similar to that of controls in the pre-biologic era but lower than controls’ in the biologic era (Table 1).

**Table 1.** Baseline characteristics of all participants in cohort.

	IBD			CD			UC		
	Case	Control	*P*-value	Case	Control	*P*-value	Case	Control	*P*-value
**All time**									
** Participants**	13 306	133 060		5955	59 611		7351	73 449	
** Death**	2156	19 095		977	8061		1179	11 034	
** Death rate (%)**	16.2	14.4		16.4	13.5		16	15	
** Male (%)**	46.4			43			49.2		
** Age at IBD diagnosis, median (IQR)**	37 (26-53)			34 (23-49)			40 (28-56)		
** Age at death, median (IQR)**	**75 (62-84)**	**76 (65-85)**	**.0038**	**72 (60-82)**	**75 (63-84)**	**<.0001**	77 (65-86)	77 (65-85)	.54
**Pre-biologic era**									
** Participants**	6646	66 427		3403	34 031		3243	32 396	
** Death**	451	4381		216	1939		235	2442	
** Death rate (%)**	6.8	6.6		6.3	5.7		7.2	7.5	
** Age at IBD diagnosis, median (IQR)**	34 (26-48)			33 (25-45)			37 (28-52)		
** Age at death, median (IQR)**	73 (60-81)	73 (61-81)	.56	70 (55.5-79)	72 (59-81)	.21	76 (62-82)	74 (63-82)	.56
**Biologic era**									
** Participants**	12 369	120 901		5448	53 310		6921	67 591	
** Death**	1705	14 714		761	6122		944	8592	
** Death rate (%)**	13.8	12.2		14	11.5		13.6	12.7	
** Age at IBD Diagnosis, median (IQR)**	37 (26-53)			34 (23-49)			40 (28-56)		
** Age at death, median (IQR)**	**76 (63**-**85)**	**77 (65**-**86)**	**.0012**	**73 (61**-**83)**	**76 (64**-**85)**	**<.0001**	78 (66-86)	78 (66-86)	.82

Comparisons of data that appear in bold are statistically significant.

Abbreviations: CD, Crohn’s disease; IBD, inflammatory bowel disease; IQR, interquartile range; UC, Ulcerative colitis.

### 3.2. All-cause mortality and cause-specific mortality

Across 1984-2019, IBD was associated with higher all-cause mortality compared with matched controls (HR 1.13, 95% CI 1.08-1.18; *P* < .0001), driven by CD (HR 1.28, 95% CI 1.19-1.37; *P* < .0001), whereas UC did not differ significantly from controls (HR 1.03, 95% CI 0.97-1.10; *P* = .30) (Table 2).

**Table 2.** Era-stratified all-cause mortality.

Era	Disease	HR	95% CI	*P*-value
**All (1984-2019)**	IBD	**1.13**	**1.08**-**1.18**	**<.0001**
	CD	**1.28**	**1.19**-**1.37**	**<.0001**
	UC	1.03	0.97-1.1	.30
**Pre-biologics (1984-2000)**	IBD	1.04	0.93-1.15	.49
	CD	1.1	0.95-1.28	.21
	UC	0.98	0.85-1.14	.83
**Biologic era (2001-2019)**	IBD	**1.16**	**1.1**-**1.22**	**<.0001**
	CD	**1.34**	**1.24**-**1.45**	**<.0001**
	UC	1.05	0.98-1.12	.21

Comparisons of data that appear in bold are statistically significant.

Abbreviations: CD, Crohn’s disease; CI, confidence interval; HR, hazard ratios; IBD, inflammatory bowel disease; UC, ulcerative colitis.

**Figure 1.**
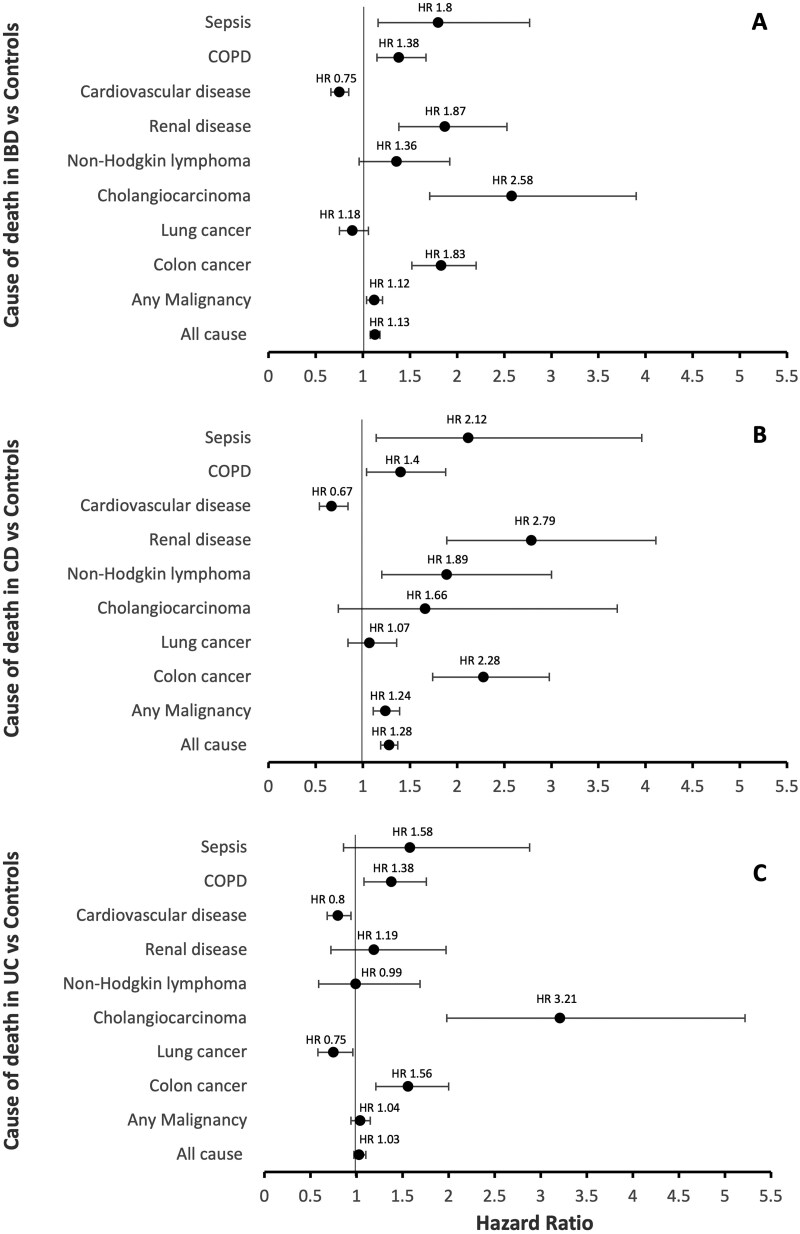
Cause-specific mortality in inflammatory bowel disease (IBD) compared with matched controls, 1984-2019. Forest plots show hazard ratios (HRs) and 95% confidence intervals for all-cause and cause-specific mortality in (A) all IBD, (B) Crohn’s disease (CD), and (C) ulcerative colitis (UC).

In CD, mortality was significantly increased relative to controls for any malignancy (HR 1.24, 95% CI 1.11-1.39; *P* = .0002), colorectal cancer (CRC) (HR 2.28, 95% CI 1.74-2.98; *P* < .0001), non-Hodgkin lymphoma (HR 1.89, 95% CI 1.20-3.00; *P* = .007), renal disease (HR 2.79, 95% CI 1.89-4.11; *P* < .0001), chronic obstructive pulmonary disease (COPD) (HR 1.40, 95% CI 1.04-1.88; *P* = .028), and sepsis (HR 2.12, 95% CI 1.14-3.96; *P* = .018). In contrast, cardiovascular disease mortality was significantly lower in CD (HR 0.67, 95% CI 0.54-0.84; *P* = .0003). Lung cancer (HR 1.07, 95% CI 0.84-1.36; *P* = .59) and cholangiocarcinoma mortality (HR 1.66, 95% CI 0.74-3.70; *P* = .22) were not significantly increased (Figure 1, Table S1).

In UC, mortality from any malignancy was not increased (HR 1.04, 95% CI 0.94-1.15; *P* = .46). However, UC was associated with significantly increased mortality from CRC (HR 1.56, 95% CI 1.21-2.00; *P* = .0006) and cholangiocarcinoma (HR 3.21, 95% CI 1.98-5.22; *P* < .0001). Among nonmalignant causes, COPD mortality was increased (HR 1.38, 95% CI 1.08-1.76; *P* = .01), whereas renal disease (HR 1.19, 95% CI 0.72-1.97; *P* = .495) and sepsis (HR 1.58, 95% CI 0.86-2.88; *P* = .14) were not significantly different from controls. Cardiovascular disease and lung cancer mortality were significantly reduced in UC (HR 0.80, 95% CI 0.68-0.94; *P* = .0065; HR 0.75, 95% CI 0.58-0.96; *P* = .023, respectively) (Figure 1, Table S1).

### 3.3. Era-specific mortality

In era-stratified analyses, crude all-cause mortality rates (per 100 000 person-years) were similar between IBD cases and matched controls in the pre-biologic era (1984-2000) (IBD: 755 vs. 764; CD: 677 vs. 631; UC: 843 vs. 918). In the biologic era (2001-2019), crude all-cause mortality rates were higher among IBD cases than controls (IBD: 1157 vs. 1040), with this difference most evident in CD (1096 vs. 902) and less pronounced in UC (1211 vs. 1167) (Figure S1 [see online supplementary material for a color version of this figure], Tables S2 and S3).

**Figure 2.**
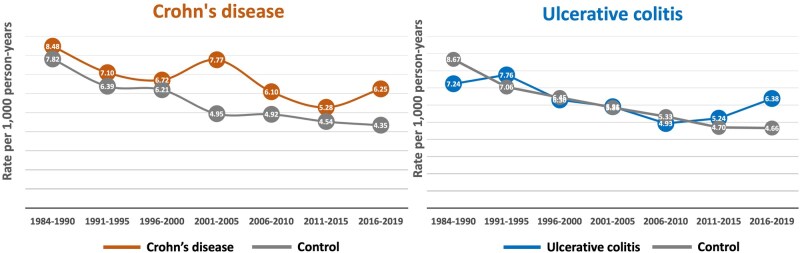
Age-standardized all-cause mortality rates per 1000 person-years are shown for Crohn’s disease and ulcerative colitis compared with matched controls across multiyear calendar intervals. Rates were standardized to the 2001 Canadian census population. Mortality rates declined over time, but the case-control gap remained more evident in Crohn’s disease than ulcerative colitis.

Age-standardized all-cause mortality rates provided similar absolute context. Across multi-year calendar intervals, rates generally declined over time in both IBD cases and matched controls. However, in CD, rates remained higher than those of matched controls in later calendar periods, whereas UC rates tracked more closely with those of controls (Figure 2, Table S4).

Consistent with these absolute rate patterns, era-stratified survival analyses demonstrated no difference in all-cause mortality between IBD and controls in the pre-biologic era (HR 1.04, 95% CI 0.93-1.15; *P* = .49), whereas a significant excess mortality emerged in the biologic era for IBD overall (HR 1.16, 95% CI 1.10-1.22; *P* < .0001), driven by CD (HR 1.34, 95% CI 1.24-1.45; *P* < .0001), while UC remained non-significant (HR 1.05, 95% CI 0.98-1.12; *P* = .21) (Table 2).

**Figure 3.**
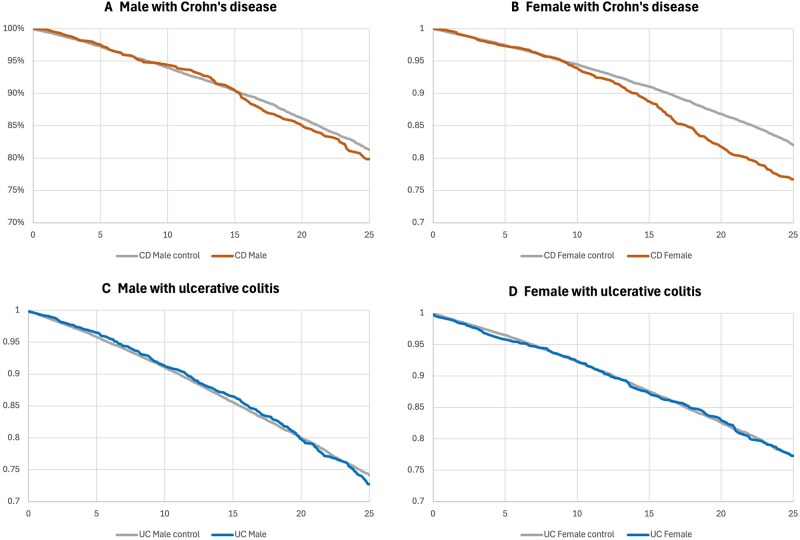
Sex-stratified Kaplan-Meier survival in Crohn’s disease and ulcerative colitis: cases vs matched controls. Survival time was measured from the date of inflammatory bowel disease (IBD) diagnosis. Panels A and B show Crohn’s disease (CD) cases and matched controls in males (A) and females (B). Panels C and D show ulcerative colitis (UC) cases and matched controls in males (C) and females (D).

Sex-stratified Kaplan–Meier curves comparing cases and controls from the date of IBD diagnosis, after excluding individuals diagnosed before 1987 (and their matched controls), showed time-dependent divergence in CD, with case-control separation emerging after approximately 10 years and more pronounced among females. This pattern was not observed in males with CD, nor in UC in either sex (Figure 3).

### 3.4. Sensitivity analyses by diagnosis era and biologic exposure

In diagnosis-era sensitivity analyses stratifying patients by diagnosis period and follow-up window, among patients diagnosed before 2001 with follow-up censored at 2000, there was no clear excess mortality in CD. Among patients diagnosed after 2001 and followed within the biologic era, estimates were imprecise because of low death counts, with no clear excess mortality in CD (HR 1.12, 95% CI 0.95-1.32). In contrast, among patients diagnosed before 2001 and followed long-term after 2000, excess mortality was apparent in CD (HR 1.42, 95% CI 1.30-1.56). In UC, mortality remained comparable to matched controls across all 3 diagnosis-era/follow-up strata. Overall, excess mortality in CD was most apparent among patients diagnosed before 2001 with long-term follow-up after 2000 (Table 3).

**Table 3.** Diagnosis-era and follow-up-period sensitivity analyses for all-cause mortality in IBD.

Era and follow-up period	Disease	HR	95% CI	*P*-value
**Diagnosed before 2001** **Follow-up censored at December 31, 2000**	IBD	1.03	0.93-1.14	.58
	CD	1.10	0.96-1.27	.18
	UC	0.97	0.84-1.11	.64
**Diagnosed after 2001** **Follow-up during biologic era**	IBD	**1.12**	**1.02**-**1.23**	**.020**
	CD	1.12	0.95-1.32	.16
	UC	1.12	0.99-1.25	.062
**Diagnosed before 2001** **Long-term follow-up after January 1, 2001**	IBD	**1.18**	**1.11**-**1.26**	**<.0001**
	CD	**1.42**	**1.30**-**1.56**	**<.0001**
	UC	1.01	0.92-1.10	.83

Comparisons of data that appear in bold are statistically significant.

Abbreviations: CD, Crohn’s disease; CI, confidence interval; HR, hazard ratios; IBD, inflammatory bowel disease; UC, ulcerative colitis.

In descriptive biologic exposure sensitivity analyses conducted during the biologic era, biologic-exposed IBD cases had higher mortality than matched controls across IBD overall (HR 1.71, 95% CI 1.38-2.11; *P* < .0001), CD (HR 1.62, 95% CI 1.25-2.08; *P* = .0002), and UC (HR 1.94, 95% CI 1.33-2.82; *P* = .0006). Among biologic-unexposed patients, excess mortality was observed in CD overall (HR 1.40, 95% CI 1.28-1.52; *P* < .0001), most clearly among females with CD (HR 1.62, 95% CI 1.45-1.81; *P* < .0001), whereas UC remained comparable to matched controls overall and by sex (Table 4).

**Table 4.** Biologic exposure sensitivity analyses for all-cause mortality during the biologic era.

Group	Disease	All			Male			Female		
		*P*-value	HR	95% CI	*P*-value	HR	95% CI	*P*-value	HR	95% CI
**Exposure**	IBD	**<.0001**	**1.71**	**1.38**-**2.11**						
	CD	**.0002**	**1.62**	**1.25**-**2.08**	.5094	1.14	0.77-1.71	**<.0001**	**2.16**	**1.55**-**3.02**
	UC	**.0006**	**1.94**	**1.33**-**2.82**	**.0015**	**2.16**	**1.34**-**3.46**	.1206	1.64	0.88-3.05
**No exposure**	IBD	**<.0001**	**1.17**	**1.11**-**1.24**						
	CD	**<.0001**	**1.40**	**1.28**-**1.52**	.0562	1.14	0.99-1.31	**<.0001**	**1.62**	**1.45**-**1.81**
	UC	.22	1.05	0.97-1.13	.7469	1.02	0.92-1.12	.1478	1.08	0.97-1.20

Comparisons of data that appear in bold are statistically significant.

Abbreviations: CD, Crohn’s disease; CI, confidence interval; HR, hazard ratios; IBD, inflammatory bowel disease; UC, ulcerative colitis.

### 3.5. Multivariate analysis for cause-specific mortality in IBD patients

In multivariable competing-risk models among IBD patients, CD was associated with higher all-cause mortality compared with UC (HR 1.23, 95% CI 1.12-1.34; *P* < .0001) (Table S5). Male sex and increasing age were independently associated with higher all-cause mortality (male vs. female: HR 1.26, 95% CI 1.15-1.37; *P* < .0001; per-year increase in age: HR 1.10, 95% CI 1.09-1.10; *P* < .0001). The biologic era was associated with lower all-cause mortality in IBD overall (HR 0.85, 95% CI 0.76-0.96; *P* = .007), and a similar reduction was observed in matched controls (HR 0.78, 95% CI 0.76-0.82; *P* < .0001).

For CRC mortality, there was no significant difference between CD and UC (HR 1.14, 95% CI 0.77-1.69; *P* = .50). CRC mortality was lower in the biologic era in IBD overall (HR 0.59, 95% CI 0.36-0.98; *P* = .040), driven by a marked reduction in UC (HR 0.27, 95% CI 0.14-0.51; *P* < .0001), whereas no reduction was observed in CD (HR 1.48, 95% CI 0.68-3.18; *P* = .32). In contrast, CRC mortality in controls did not change by era (HR 1.01, 95% CI 0.45-2.27; *P* = .97).

For cardiovascular disease mortality, the biologic era was associated with lower cardiovascular mortality in IBD overall (HR 0.61, 95% CI 0.48-0.77; *P* < .0001), with consistent reductions in both CD (HR 0.69, 95% CI 0.48-0.99; *P* = .045) and UC (HR 0.57, 95% CI 0.42-0.77; *P* = .0003). However, a similar reduction was also observed in controls (HR 0.53, 95% CI 0.43-0.65; *P* < .0001).

Lung cancer mortality was higher in CD compared with UC (HR 1.59, 95% CI 1.13-2.24; *P* = .008), and there was no evidence of an era effect on lung cancer mortality in the overall IBD cohort (HR 0.98, 95% CI 0.62-1.55; *P* = .92). In controls, however, lung cancer mortality declined in the biologic era (HR 0.53, 95% CI 0.34-0.83; *P* = .005). Respiratory-related mortality did not differ between CD and UC (HR 1.13, 95% CI 0.85-1.51; *P* = .40), and no biologic-era effect was observed in IBD (HR 1.10, 95% CI 0.75-1.61; *P* = .63). Non-Hodgkin lymphoma mortality tended to be higher in CD than UC (HR 1.98, 95% CI 0.97-4.03; *P* = .062) but there was no biologic-era effect within IBD (HR 1.58, 95% CI 0.62-4.01; *P* = .34), whereas controls demonstrated a significant reduction in lymphoma mortality in the biologic era (HR 0.29, 95% CI 0.11-0.76; *P* = .012).

Finally, IBD-related mortality showed the clearest disease-subtype separation, with substantially higher mortality in CD compared with UC (HR 2.96, 95% CI 1.96-4.46; *P* < .0001). IBD-related mortality was markedly reduced in the biologic era in IBD overall (HR 0.37, 95% CI 0.23-0.59; *P* < .0001), with similar reductions in the biologic era in CD (HR 0.38, 95% CI 0.21-0.67; *P* = .0008) and UC (HR 0.32, 95% CI 0.14-0.72; *P* = .006).

## 4. Discussion

IBD has been associated with an increased long-term risk of mortality since the 1950s, although the magnitude of excess risk has varied by geography, disease subtype, and calendar period.^13^^,^^15–33^ Across cohorts, leading contributors to death most consistently included digestive disease, malignancy, respiratory disease, cardiovascular disease, and infections.^15^^,^^16^^,^^20–33^ With biologic therapy and earlier steroid-sparing strategies now central to care, a key question is whether modern management has narrowed mortality differences between IBD and the background population.

In this population-based matched cohort (1984-2019), all-cause mortality in CD was 28% higher than in controls, whereas UC mortality did not differ from controls. After adjustment for age, sex, and era, CD remained independently associated with higher all-cause mortality than UC (HR 1.23, 95% CI 1.12-1.34; *P* < .0001). Era‑stratified analyses showed comparable mortality between IBD and controls in the pre‑biologic era (1984-2000), but excess mortality emerged in the biologic era (2001-2019), driven by CD, while UC remained similar to controls.

Our pre-biologic findings—particularly the absence of a significant mortality excess in UC—are consistent with earlier syntheses reporting minimal or no increase in UC mortality across mixed historical eras (HR 1.1, 95% CI 0.9-1.2; *P* = .42).^17^ In contrast, CD mortality has more consistently been elevated in prior work, often around SMR 1.4-1.5.^14^^,^^18^^,^^19^ Notably, in our pre-biologic analysis, CD mortality was not significantly higher than controls (HR 1.10, 95% CI 0.95-1.28). This finding was further supported by the diagnosis-era sensitivity analysis restricted to patients diagnosed before 2001 with follow-up censored at 2000, which also showed no clear excess mortality in CD.

In the biologic era, excess mortality persisted and was most clearly concentrated in CD, aligning with contemporary population‑based reports from North America, the UK/Europe, and New Zealand, as well as recent meta‑analytic estimates indicating ongoing mortality differentials in CD and, to a lesser extent, UC.^20^^,^^21^^,^^29^^,^^31^^,^^33^ However, not all mixed-era inception cohorts have shown uniform excess mortality. In the Olmsted County inception cohort (1970-2016), UC mortality was lower than expected, and CD mortality was not significantly increased.^42^ Nationwide data from the Faroe Islands (1966-2020)^34^ and the Norwegian IBSEN inception cohort (1990-2020)^43^ similarly reported no significant increase in mortality in CD or UC compared with controls. Collectively, these findings underscore heterogeneity across settings, which may reflect differences in cohort composition, disease severity, analytic methods, secular trends in background survival, and limited power in smaller cohorts.

Because excess mortality in CD was most apparent during the biologic era, we performed individual-level biologic exposure sensitivity analyses to contextualize this calendar-era finding. Mortality was higher among biologic-exposed patients across IBD subtypes, a pattern likely reflecting confounding by indication and greater disease severity rather than a causal biologic effect. Among biologic-unexposed patients, excess mortality was most evident in females with CD, suggesting that the CD mortality signal was not limited to biologic-exposed patients.

Interpretation of era effects should also account for population-wide improvements in survival. In our models, the biologic era was associated with lower all-cause mortality in IBD (HR 0.85, 95% CI 0.76-0.96; *P* = .007), but a similar decline was observed in controls (HR 0.78, 95% CI 0.76-0.82; *P* < .0001), suggesting that part of the apparent “era benefit” reflects broader secular improvements rather than IBD-specific effects alone. This interpretation was supported by age-standardized all-cause mortality rates, which generally declined over time in both IBD cases and matched controls, although rates remained higher in CD than controls in later calendar periods. Canadian and global trend analyses similarly report declining mortality over time for CD and UC,^20^^,^^44^ although some data suggest more recent increases around the COVID‑19 period.^45^

Diagnosis-era sensitivity analyses further clarified the timing of the CD mortality signal. Excess mortality became apparent among patients diagnosed before 2001 who were followed long-term after 2000, but it was not evident among incident post-2001 CD cases. Sex-stratified Kaplan–Meier curves further showed time-dependent divergence in CD, with separation from matched controls emerging after approximately 10 years in females, while male CD and UC in both sexes remained largely comparable to controls. The biologic-unexposed sensitivity analysis showed a similar pattern, with excess mortality most evident in females with CD. Prior evidence regarding sex differences in IBD mortality is mixed, but several analyses have reported higher mortality in women with CD.^18^^,^^28^^,^^45^ These findings support sex as a potential effect modifier of late mortality risk in CD.

Several biologically plausible pathways could contribute to this pattern. Proposed mechanisms include X chromosome enrichment of IBD-associated variants and sex-dependent differences in mucosal immune activation,^46^^,^^47^ as well as estrogen receptor signaling effects on barrier function and immune homeostasis, which may change with aging.^47^ In addition, women with IBD may experience a higher burden of extraintestinal manifestations and may be less likely to persist with immunomodulators and biologics, with greater reliance on prolonged systemic corticosteroids, an established predictor of serious infection and mortality, particularly in older patients.^36^^,^^37^^,^^47^^,^^48^ These hypotheses provide a coherent framework for the late‑emerging divergence observed among females with CD, but require validation with clinical phenotype and treatment‑exposure data.

In crude cause‑of‑death tabulations, malignancy accounted for the largest proportion of deaths among individuals with IBD, followed by cardiovascular, respiratory, renal, and infection‑related causes (Figure S1 [see online supplementary material for a color version of this figure] and Tables S2 and S3). However, absolute rankings can obscure the drivers of excess risk. In competing‑risk analyses versus matched controls, cardiovascular mortality was not increased and appeared lower (Table S1), highlighting a divergence between the common causes of death and those that account for the mortality gap. In our cohort, excess mortality was more closely linked to specific malignancies, infections, and respiratory disease.

Across the full study period, IBD was associated with increased CRC mortality (HR 1.83, 95% CI 1.52-2.20), driven predominantly by CD (HR 2.28, 95% CI 1.74-2.98). The excess in UC was more modest (HR 1.56, 95% CI 1.21-2.00) and was not clearly different from CD after adjustment for sex, age, and era. This contrasts with historical patterns in which UC was often the dominant contributor to colitis‑associated CRC mortality^13^^,^^15^^,^^29^ but is consistent with Canadian population data showing a stronger signal in CD than UC.^20^ Era-stratified analyses clarify this divergence: in the biologic era, CRC-related mortality declined substantially in UC, with no corresponding reduction in CD or in controls. Earlier Manitoba data (1984-1997) showed elevated CRC incidence in both UC and CD,^49^ whereas more recent studies and meta‑analyses show marked reductions in UC CRC risk and mortality in contemporary practice, likely reflecting improved therapy and surveillance.^50–53^

The decline in UC CRC mortality likely reflects the impact of structured dysplasia surveillance program. Risk-stratified surveillance is supported for patients with extensive colitis, prior low-grade dysplasia, concomitant PSC, strictures, and a family history of CRC.^52^^,^^54^ Because surveillance in UC is triggered by disease duration and IBD-specific risk factors, it enables detection and treatment of premalignant lesions or early cancers before progression to fatal disease.^52^^,^^55^ In contrast, guideline consensus and the evidence base for surveillance in Crohn’s colitis have historically been weaker, in part because foundational studies were UC-centric.^54^^,^^56^^,^^57^ Consequently, some patients with CD, particularly those without clearly documented extensive colitis, may default to average-risk, age-based screening rather than duration-based IBD surveillance. Supporting this concern, a large Scandinavian study reported higher CRC mortality among patients with CD who developed CRC than in reference individuals with CRC, even after adjustment for tumor stage. A similar stage at diagnosis may indicate that surveillance and/or downstream management pathways have not translated into meaningful mortality gains in CD.^57^

Multiple clinical and biological features may impede comparable progress in CD. Colitis-associated carcinogenesis differs from sporadic CRC, with early p53 alterations preceding APC mutations and a strong dependence on chronic inflammation.^58^^,^^59^ The segmental, transmural nature of CD may lead to a higher cumulative inflammatory burden over time, sustaining carcinogenic pressure despite improved medical control.^52^^,^^59^ In addition, colonic strictures—more common in CD—are both high‑risk sites and practical barriers to complete colonoscopic surveillance, contributing to interval cancers.^54^^,^^60^ Finally, the rising incidence of early-onset colorectal cancer, together with age-based screening paradigms for average-risk populations (now recommending initiation at age 45 years),^61^ may blunt mortality gains in CD when patients who meet duration- or inflammation-based IBD risk criteria are not enrolled in IBD-specific surveillance programs. This concern is amplified in individuals diagnosed with CD before age 40 years—an exposure associated with higher CRC risk—supporting the need for earlier, duration- and risk-stratified surveillance rather than reliance on age thresholds alone.^57^

We observed an excess of cholangiocarcinoma mortality concentrated in UC, consistent with the epidemiology of PSC, which is far more prevalent in UC than CD and is a major risk factor for hepatobiliary cancers.^15^^,^^25^^,^^62–64^ The limited number of events precluded robust assessment of era-specific temporal trends. We also identified a CD-predominant increase in lymphoma-related mortality. Similar to previous data, there was approximately twice the risk of death in lymphoma in CD compared with controls.^15^^,^^16^^,^^20^ This aligns with extensive evidence linking lymphoma risk in IBD to immunosuppressive therapy, particularly long-term thiopurine use, especially in combination with anti-TNF agents.^65–68^ Although absolute risks remain small, these findings reinforce the importance of treatment‑risk stratification and vigilant monitoring in high‑risk groups, including younger males with CD.^69^

In our cohort, cardiovascular disease mortality was not increased and appeared lower in IBD than in controls, despite robust evidence linking IBD to a higher incidence of myocardial infarction and stroke, particularly among younger patients and women.^70^ While 2 large cohorts have reported modestly increased cardiovascular mortality (eg, Sweden adult-onset IBD and the IBSEN inception cohort),^25^^,^^43^ this apparent paradox—higher arterial event risk but neutral or even lower CVD mortality—has been observed across several population-based cohorts and meta-analyses, which typically report CVD-related SMRs close to unity or slightly below for both UC and CD.^15^^,^^20^^,^^28^^,^^71^ A recent Faroese population-based cohort similarly suggested a significantly lower risk of cardiovascular death in IBD (SIR 0.7, 95% CI 0.50-0.93), highlighting persistent geographic heterogeneity.^34^ The drivers of these differences remain uncertain. One plausible explanation is competing risks, whereby malignancy, digestive disease, and sepsis contribute disproportionately to mortality in IBD, while arterial events may occur later, be more treatable, or be less frequently coded as the underlying cause of death. In our data, CVD mortality declined in the biologic era in both IBD and controls, consistent with secular improvements in primary prevention and acute cardiovascular care.

Consistent with other population-based studies, infections, respiratory disease, and renal disease were important contributors to excess mortality in IBD, particularly in CD. Serious infections—including sepsis, intra-abdominal and postoperative infections, pneumonia, and opportunistic infections—featured prominently as underlying or immediate causes, ­especially among older patients and those with prior surgery or prolonged corticosteroid exposure and combination immunosuppressive therapy, mirroring large registry and cohort data showing clearly elevated infection-related mortality in IBD compared with the background population.^15^^,^^16^^,^^20^^,^^27^^,^^36^^,^^43^^,^^63^^,^^72^ Respiratory diseases, mainly COPD, also contributed meaningfully to mortality, aligning with earlier population-based work and meta-analyses reporting a 1.5- to 2-fold higher risk of pulmonary-related death in IBD than in the general population.^15^^,^^25^ Potential mechanistic links include chronic smoking (particularly in CD), systemic inflammation, drug-induced lung injury, and under-recognized extraintestinal pulmonary involvement.^73^ Renal causes accounted for a smaller proportion of deaths, but the excess risk was most evident in CD, in keeping with growing evidence that chronic kidney disease is increasingly prevalent as a comorbidity in IBD.^74–77^ In a nationwide cohort, Park et al. reported that, after adjustment for age, sex, residence, income, comorbidities, and medication use, all-cause mortality in patients with end-stage renal disease was 2.79-fold higher in CD and 1.98-fold higher in UC compared with controls.^74^

IBD-related deaths accounted for only 5% of all deaths in our cohort; however, IBD-related mortality declined substantially in the biologic era in both CD and UC. IBD-related mortality fell by almost two-thirds in the biologic era (HR 0.37, 95% CI 0.23-0.59 for IBD overall; 0.38 for CD; 0.32 for UC). This decline likely reflects broader advances in IBD care, including steroid-sparing treatment strategies, earlier intervention, closer monitoring, tighter disease control, and improvements in surgical and perioperative management. Among U.S. Medicaid/Medicare beneficiaries with IBD, anti-TNF initiation in CD was associated with lower mortality than prolonged corticosteroid use (21.4 vs. 30.1 per 1000 patient-years; OR 0.78, 95% CI 0.65-0.93).^40^ Similarly, Veterans Health Administration data demonstrated lower mortality with anti-TNF therapy compared with chronic corticosteroid exposure in both UC and CD, particularly within 90-270 days after anti-TNF initiation.^78^ In parallel, national surgical datasets from the biologic era document declining operative rates and improved postoperative outcomes: IBD surgery decreased from 10% to 8.8% of admissions for CD and from 7.7% to 7.5% for UC, while 30-day mortality after colectomy in UC fell from 5.8% to 2.3%.^79^ However, because calendar era does not capture individual treatment exposure, these findings should be interpreted as population-level era trends rather than direct evidence of causal biologic treatment effects.

Clinically, these findings support a broader prevention framework for long-term mortality risk in IBD, particularly CD. Beyond symptom control, prevention should include timely effective steroid-sparing therapy to control inflammation and reduce prolonged corticosteroid exposure; risk-stratified CRC surveillance in Crohn’s colitis; vaccination and infection prevention; renal function monitoring; smoking cessation; and respiratory infection prevention.

The strengths of this study include its large, population-based design with nearly 40 years of follow-up, which minimizes selection bias, enhances generalizability, and enables estimation of rare but clinically important outcomes (eg, cholangiocarcinoma and lymphoma). To our knowledge, this is the first cohort to systematically compare cause-specific mortality in IBD versus matched non-IBD controls stratified by calendar era (pre-biologic vs. biologic), providing a framework to evaluate how changes in IBD management and broader healthcare improvements may have reshaped mortality patterns over time. The use of competing-risk methods in both cases and controls further supports a more nuanced assessment of cause-specific mortality beyond proportional comparisons alone.

Several limitations warrant consideration. Our administrative data lack information on CD phenotype, particularly colonic involvement; therefore, CRC mortality estimates in CD may have been diluted by inclusion of patients with isolated small bowel disease together with those with Crohn’s colitis. For other cause-specific outcomes, we also lacked information on disease activity, inflammatory burden, baseline severity, frailty, smoking history and some details about comorbidities that may influence competing causes of death, allowing residual confounding. Calendar era did not capture individual biologic exposure, and biologic exposure analyses were subject to confounding by indication; these findings should therefore not be interpreted as causal biologic treatment effects. Despite these limitations, the population-based design, matched controls, long follow-up, age-standardized rates, sensitivity analyses, and competing-risk methods strengthen interpretation of IBD-specific mortality patterns.

In conclusion, excess mortality in IBD was most evident in CD, appeared to emerge with longer follow-up, especially in females, and was concentrated in selected causes including colorectal cancer, sepsis, respiratory disease, and renal disease. Mortality in UC was largely comparable to matched controls. These findings highlight the need for sustained long-term risk monitoring and prevention in high-risk CD populations.

## Supplementary Material

jjag105_Supplementary_Data

## Data Availability

The data underlying this study contain sensitive personal health information and cannot be shared publicly due to patient privacy and ethical restrictions. All results generated for this study are presented in this manuscript and Supplementary Material. No additional data or study materials are available.
