# Supplementary material for: All-cause and cause-specific mortality in inflammatory bowel disease across the biologic era: a population-based matched cohort study
Source: J Crohns Colitis. 2026 Jul 13;20(7):jjag105. doi: 10.1093/ecco-jcc/jjag105 (PMC13361712; doi:10.1093/ecco-jcc/jjag105)

**Supplementary data**

| **Table S1. All-cause mortality and cause-specific mortality in IBD patients across 1984-2019** | | | | | | | | | | |
| --- | --- | --- | --- | --- | --- | --- | --- | --- | --- | --- |
| Variables | | **IBD** | | | **CD** | | | **UC** | | |
|  |  | Death | P value | HR  (95%CI) | Death | P value | HR  (95%CI) | Death | P value | HR  (95%CI) |
| **All-cause** | Case | 2156 | **<.0001** | **1.13**  **(1.08,1.18)** | 977 | **<.0001** | **1.28**  **(1.19,1.37)** | 1179 | 0.357 | 1.03  (0.97,1.10) |
|  | Control | 19095 |  |  | 8061 |  |  | 11034 |  |  |
| **Any Malignancy** | Case | 656 | **0.0028** | **1.12**  **(1.04,1.21)** | 305 | **0.0002** | **1.24**  **(1.11,1.39)** | 351 | 0.46 | 1.04  (0.94,1.15) |
|  | Control | 5630 |  |  | 2397 |  |  | 3233 |  |  |
| **Colon cancer** | Case | 114 | **<.0001** | **1.83**  **(1.52,2.20)** | 54 | **<.0001** | **2.28**  **(1.74,2.98)** | 60 | **0.0006** | **1.56**  **(1.21,2.00)** |
|  | Control | 597 |  |  | 234 |  |  | 363 |  |  |
| **Lung cancer** | Case | 128 | 0.18 | 0.89  (0.75,1.06) | 67 | 0.59 | 1.07  (0.84,1.36) | 61 | **0.023** | **0.75**  **(0.58,0.96)** |
|  | Control | 1401 |  |  | 614 |  |  | 787 |  |  |
| **Cholangiocarcinoma** | Case | 23 | **<.0001** | **2.58**  **(1.71,3.90)** | 6 | 0.22 | 1.66  (0.74,3.7) | 17 | **<.0001** | **3.21**  **(1.98,5.22)** |
|  | Control | 85 |  |  | 36 |  |  | 49 |  |  |
| **Non-Hodgkin Lymphoma** | Case | 32 | 0.085 | 1.36  (0.96,1.92) | 18 | **0.007** | **1.89**  **(1.20,3.00)** | 14 | 0.99 | 0.995  (0.59,1.69) |
|  | Control | 230 |  |  | 94 |  |  | 136 |  |  |
| **Others malignancy** | Case | 359 | 0.47 | 1.04  (0.94-1.15) | 160 | 0.24 | 1.097  (0.944-1.28) | 199 | 0.97 | 0.997  (0.87-1.15) |
|  | Control | 3317 |  |  | 1419 |  |  | 1898 |  |  |
| **Renal disease** | Case | 41 | **<.0001** | **1.87**  **(1.38,2.53)** | 26 | **<.0001** | **2.79**  **(1.89,4.11)** | 15 | 0.495 | 1.19  (0.72,1.97) |
|  | Control | 209 |  |  | 90 |  |  | 119 |  |  |
| **Cardiovascular disease** | Case | 225 | **<.0001** | **0.75**  **(0.66,0.85)** | 83 | **0.0003** | **0.67**  **(0.54,0.84)** | 142 | **0.0065** | **0.8**  **(0.68,0.94)** |
|  | Control | 2910 |  |  | 1177 |  |  | 1733 |  |  |
| **COPD** | Case | 109 | **0.0007** | **1.38**  **(1.15,1.67)** | 42 | **0.028** | **1.4**  **(1.04,1.88)** | 67 | **0.01** | **1.38**  **(1.08,1.76)** |
|  | Control | 781 |  |  | 299 |  |  | 482 |  |  |
| **Sepsis** | Case | 21 | **0.0082** | **1.8**  **(1.16,2.77)** | 10 | **0.018** | **2.12**  **(1.14,3.96)** | 11 | 0.14 | 1.58  (0.86,2.88) |
|  | Control | 112 |  |  | 46 |  |  | 66 |  |  |
| Comparisons of data that appear in bold are statistically significant.  CD, Crohn’s disease; UC, Ulcerative colitis; IBD, Inflammatory bowel disease; HR, Hazard ratios; CI, Confidence interval; COPD, Chronic obstructive pulmonary disease | | | | | | | | | | |

| **Table S2. Crude cause-specific mortality in IBD patients and matched controls during the pre-biologic era (1984–2000)** | | | | | | | |
| --- | --- | --- | --- | --- | --- | --- | --- |
| Variables | | **IBD** | | **CD** | | **UC** | |
|  |  | Death | Rate per 100000 PYs | Death | Rate per 100000 PYs | Death | Rate per 100000 PYs |
| **All-cause** | Case | 451 | 755 | 216 | 677 | 235 | 843 |
|  | Control | 4381 | 764 | 1939 | 631 | 2442 | 918 |
| **Any Malignancy** | Case | 135 | 226 | 59 | 185 | 76 | 273 |
|  | Control | 1307 | 228 | 579 | 188 | 728 | 274 |
| **Colon cancer** | Case | 33 | 55 | 11 | 34 | 22 | 79 |
|  | Control | 129 | 23 | 56 | 18 | 73 | 27 |
| **Lung cancer** | Case | 27 | 45 | 12 | 38 | 15 | 54 |
|  | Control | 363 | 63 | 150 | 49 | 213 | 80 |
| **Cholangiocarcinoma** | Case | Too few | | | | | |
|  | Control |  |  |  |  |  |  |
| **Non-Hodgkin Lymphoma** | Case | 6 | 10 |  |  |  |  |
|  | Control | 61 | 11 |  |  |  |  |
| **Others malignancy** | Case | 67 | 112 | 32 | 100 | 35 | 126 |
|  | Control | 746 | 130 | 348 | 113 | 398 | 150 |
| **Renal disease** | Case | Too few | | | | | |
|  | Control |  |  |  |  |  |  |
| **Cardiovascular disease** | Case | 67 | 112 | 27 | 85 | 40 | 144 |
|  | Control | 899 | 157 | 372 | 121 | 527 | 198 |
| **COPD** | Case | 19 | 32 |  |  |  |  |
|  | Control | 122 | 21 |  |  |  |  |
| **Sepsis** | Case | Too few | | | | | |
|  | Control |  |  |  |  |  |  |
| CD, Crohn’s disease; UC, Ulcerative colitis; IBD, Inflammatory bowel disease; COPD, Chronic obstructive pulmonary disease; PYs, Person years | | | | | | | |

| **Table S3. Crude cause-specific mortality in IBD patients and matched controls during the biologic era (2001-2019)** | | | | | | | |
| --- | --- | --- | --- | --- | --- | --- | --- |
| Variables | | **IBD** | | **CD** | | **UC** | |
|  |  | Death | Rate per 100000 PYs | Death | Rate per 100000 PYs | Death | Rate per 100000 PYs |
| **All-cause** | Case | 1705 | 1157 | 761 | 1096 | 944 | 1211 |
|  | Control | 14714 | 1040 | 6122 | 902 | 8592 | 1167 |
| **Any Malignancy** | Case | 521 | 354 | 246 | 354 | 275 | 353 |
|  | Control | 4323 | 306 | 1818 | 268 | 2505 | 340 |
| **Colon cancer** | Case | 81 | 55 | 43 | 62 | 38 | 49 |
|  | Control | 468 | 33 | 178 | 26 | 290 | 39 |
| **Lung cancer** | Case | 101 | 69 | 55 | 79 | 46 | 59 |
|  | Control | 1038 | 73 | 464 | 68 | 574 | 78 |
| **Cholangiocarcinoma** | Case | 21 | 14 | 6 | 9 | 15 | 19 |
|  | Control | 77 | 5 | 32 | 5 | 45 | 6 |
| **Non-Hodgkin Lymphoma** | Case | 26 | 18 | 14 | 20 | 12 | 15 |
|  | Control | 169 | 12 | 73 | 11 | 96 | 13 |
| **Others malignancy** | Case | 292 | 198 | 128 | 184 | 164 | 210 |
|  | Control | 2571 | 182 | 1071 | 158 | 1500 | 204 |
| **Renal disease** | Case | 40 | 27 | 25 | 36 | 15 | 19 |
|  | Control | 199 | 14 | 89 | 13 | 110 | 15 |
| **Cardiovascular disease** | Case | 158 | 107 | 56 | 81 | 102 | 131 |
|  | Control | 2011 | 142 | 805 | 119 | 1206 | 164 |
| **COPD** | Case | 90 | 61 | 37 | 53 | 53 | 68 |
|  | Control | 659 | 47 | 256 | 38 | 403 | 55 |
| **Sepsis** | Case | 18 | 12 | 9 | 13 | 9 | 12 |
|  | Control | 102 | 7 | 40 | 6 | 62 | 8 |
| CD, Crohn’s disease; UC, Ulcerative colitis; IBD, Inflammatory bowel disease; COPD, Chronic obstructive pulmonary disease; PYs, Person years | | | | | | | |
|  | | | | | | | |

| **Table S4. Age-standardised all-cause mortality rates per 1,000 person-years by IBD subtype and calendar period** | | | | | | | | |
| --- | --- | --- | --- | --- | --- | --- | --- | --- |
| Year | **Crohn’s disease** | | | | **Ulcerative colitis** | | | |
|  | Std Rate per 1000 | | Rate Ratio (95% CI) | P | Std Rate per 1000 | | Rate Ratio (95% CI) | P |
|  | Case | Ctrl |  |  | Case | Ctrl |  |  |
| 1984-1990 | 8.48 | 7.82 | 1.09 (0.79-1.48) | 0.61 | 7.24 | 8.67 | 0.83 (0.60-1.16) | 0.28 |
| 1991-1995 | 7.10 | 6.39 | 1.11 (0.86-1.44) | 0.42 | 7.76 | 7.06 | 1.10 (0.88-1.38) | 0.41 |
| 1996-2000 | 6.72 | 6.21 | 1.08 (0.88-1.33) | 0.46 | 6.30 | 6.45 | 0.98 (0.80-1.20) | 0.82 |
| 2001-2005 | 7.77 | 4.95 | 1.57 (1.33-1.86) | <.0001 | 5.91 | 5.86 | 1.01 (0.84-1.21) | 0.92 |
| 2006-2010 | 6.10 | 4.92 | 1.24 (1.05-1.46) | 0.010 | 4.93 | 5.33 | 0.92 (0.78-1.10) | 0.37 |
| 2011-2015 | 5.28 | 4.54 | 1.16 (0.98-1.38) | 0.08 | 5.24 | 4.70 | 1.11 (0.95-1.30) | 0.18 |
| 2016-2019 | 6.25 | 4.35 | 1.44 (1.22-1.70) | <.0001 | 6.38 | 4.66 | 1.37 (1.17-1.60) | <.0001 |
| Rates were standardised to the 2001 Canadian Census population and are reported per 1000 person-years | | | | | | | | |

| **Table S5. Multivariable models for all-cause and cause-specific mortality among IBD cases** | | | | | | |
| --- | --- | --- | --- | --- | --- | --- |
| **Cause of death** | **Disease** | **Death** | **Predictor** | **HR** | **95% CI** | **P value** |
| **All cause** | IBD | 2156 | CD vs UC | 1.23 | 1.12-1.34 | <.0001 |
|  |  |  | Male vs Female | 1.26 | 1.15-1.37 | <.0001 |
|  |  |  | Age | 1.10 | 1.09-1.10 | <.0001 |
|  |  |  | Biologic Era | 0.85 | 0.76-0.96 | 0.007 |
|  | CD | 977 | Male vs Female | 1.07 | 0.95-1.22 | 0.27 |
|  |  |  | Age | 1.09 | 1.09-1.10 | <.0001 |
|  |  |  | Biologic Era | 0.84 | 0.71- 1.01 | 0.057 |
|  | UC | 1179 | Male vs Female | 1.43 | 1.23-1.60 | <.0001 |
|  |  |  | Age | 1.10 | 1.1-1.11 | <.0001 |
|  |  |  | Biologic Era | 0.84 | 0.72-0.98 | 0.027 |
|  | Control | 19095 | Male vs Female | 1.54 | 1.49-1.58 | <.0001 |
|  |  |  | Age | 1.10 | 1.099-1.101 | <.0001 |
|  |  |  | Biologic Era | 0.78 | 0.76-0.82 | <.0001 |
| **Colorectal Cancer** | IBD | 114 | CD vs UC | 1.14 | 0.77-1.69 | 0.50 |
|  |  |  | Male vs Female | 1.92 | 1.31-2.80 | 0.0007 |
|  |  |  | Age | 1.03 | 1.02-1.04 | <.0001 |
|  |  |  | Biologic Era | 0.59 | 0.36-0.98 | 0.040 |
|  | CD | 54 | Male vs Female | 2.04 | 1.19-3.47 | 0.009 |
|  |  |  | Age | 1.03 | 1.02-1.05 | <.0001 |
|  |  |  | Biologic Era | 1.48 | 0.68-3.18 | 0.32 |
|  | UC | 60 | Male vs Female | 1.78 | 1.05-3.02 | 0.032 |
|  |  |  | Age | 1.03 | 1.01-1.04 | <.0001 |
|  |  |  | Biologic Era | 0.27 | 0.14-0.51 | <.0001 |
|  | Control | 52 | Male vs Female | 1.06 | 0.61-1.83 | 0.85 |
|  |  |  | Age | 1.04 | 1.03-1.05 | <.0001 |
|  |  |  | Biologic Era | 1.01 | 0.45-2.27 | 0.97 |
| **Lung Cancer** | IBD | 128 | CD vs UC | 1.59 | 1.13-2.24 | 0.0079 |
|  |  |  | Male vs Female | 1.31 | 0.92-1.86 | 0.13 |
|  |  |  | Age | 1.05 | 1.05-1.06 | <.0001 |
|  |  |  | Biologic Era | 0.98 | 0.62-1.55 | 0.92 |
|  | CD | 67 | Male vs Female | 0.94 | 0.57-1.55 | 0.80 |
|  |  |  | Age | 1.05 | 1.04-1.06 | <.0001 |
|  |  |  | Biologic Era | 1.08 | 0.55-2.15 | 0.82 |
|  | UC | 61 | Male vs Female | 1.93 | 1.13-3.27 | 0.016 |
|  |  |  | Age | 1.06 | 1.05-1.07 | <.0001 |
|  |  |  | Biologic Era | 0.82 | 0.45-1.51 | 0.53 |
|  | Control | 136 | Male vs Female | 1.22 | 0.87-1.71 | 0.25 |
|  |  |  | Age | 1.05 | 1.04-1.05 | <.0001 |
|  |  |  | Biologic Era | 0.53 | 0.34-0.83 | 0.0053 |
| **Non-Hodgkin Lymphoma** | IBD | 32 | CD vs UC | 1.98 | 0.97-4.03 | 0.062 |
|  |  |  | Male vs Female | 1.36 | 0.69-2.68 | 0.38 |
|  |  |  | Age | 1.05 | 1.04-1.07 | <.0001 |
|  |  |  | Biologic Era | 1.58 | 0.62-4.01 | 0.34 |
|  | CD | 18 | Male vs Female | 1.26 | 0.5-3.17 | 0.62 |
|  |  |  | Age | 1.06 | 1.04-1.07 | <.0001 |
|  |  |  | Biologic Era | 1.41 | 0.43-4.63 | 0.57 |
|  | UC | 14 | Male vs Female | 1.49 | 0.53-4.2 | 0.45 |
|  |  |  | Age | 1.05 | 1.02-1.07 | <.0001 |
|  |  |  | Biologic Era | 1.91 | 0.41-8.84 | 0.41 |
|  | Control | 28 | Male vs Female | 0.89 | 0.41-1.92 | 0.76 |
|  |  |  | Age | 1.07 | 1.05-1.09 | <.0001 |
|  |  |  | Biologic Era | 0.29 | 0.11-0.76 | 0.012 |
| **Cardiovascular disease** | IBD | 504 | CD vs UC | 1.05 | 0.88-1.27 | 0.58 |
|  |  |  | Male vs Female | 1.22 | 1.02-1.46 | 0.027 |
|  |  |  | Age | 1.08 | 1.07-1.08 | <.0001 |
|  |  |  | Biologic Era | 0.61 | 0.48-0.77 | <.0001 |
|  | CD | 205 | Male vs Female | 1.07 | 0.8-1.42 | 0.66 |
|  |  |  | Age | 1.08 | 1.07-1.09 | <.0001 |
|  |  |  | Biologic Era | 0.69 | 0.48-0.99 | 0.045 |
|  | UC | 299 | Male vs Female | 1.34 | 1.06-1.69 | 0.013 |
|  |  |  | Age | 1.08 | 1.07-1.09 | <.0001 |
|  |  |  | Biologic Era | 0.57 | 0.42-0.77 | 0.0003 |
|  | Control | 607 | Male vs Female | 1.56 | 1.32-1.83 | <.0001 |
|  |  |  | Age | 1.07 | 1.07-1.08 | <.0001 |
|  |  |  | Biologic Era | 0.53 | 0.43-0.65 | <.0001 |
| **Respiratory** | IBD | 197 | CD vs UC | 1.13 | 0.85-1.51 | 0.40 |
|  |  |  | Male vs Female | 1.27 | 0.95-1.68 | 0.11 |
|  |  |  | Age | 1.07 | 1.07-1.08 | <.0001 |
|  |  |  | Biologic Era | 1.10 | 0.75-1.61 | 0.63 |
|  | CD | 82 | Male vs Female | 1.21 | 0.77-1.9 | 0.40 |
|  |  |  | Age | 1.08 | 1.07-1.09 | <.0001 |
|  |  |  | Biologic Era | 0.85 | 0.47-1.52 | 0.57 |
|  | UC | 115 | Male vs Female | 1.31 | 0.9-1.91 | 0.16 |
|  |  |  | Age | 1.07 | 1.06-1.08 | <.0001 |
|  |  |  | Biologic Era | 1.27 | 0.75-2.12 | 0.37 |
|  | Control | 132 | Male vs Female | 1.52 | 1.07-2.16 | 0.020 |
|  |  |  | Age | 1.09 | 1.08-1.1 | <.0001 |
|  |  |  | Biologic Era | 0.65 | 0.41-1.02 | 0.059 |
| **Inflammatory bowel disease** | IBD | 101 | CD vs UC | 2.96 | 1.96-4.46 | <.0001 |
|  |  |  | Male vs Female | 0.87 | 0.58-1.31 | 0.51 |
|  |  |  | Age | 1.07 | 1.06-1.08 | <.0001 |
|  |  |  | Biologic Era | 0.37 | 0.23-0.59 | <.0001 |
|  | CD | 68 | Male vs Female | 0.82 | 0.49-1.36 | 0.44 |
|  |  |  | Age | 1.06 | 1.05-1.07 | <.0001 |
|  |  |  | Biologic Era | 0.38 | 0.21-0.67 | 0.0008 |
|  | UC | 33 | Male vs Female | 1.02 | 0.52-2.01 | 0.95 |
|  |  |  | Age | 1.09 | 1.07-1.12 | <.0001 |
|  |  |  | Biologic Era | 0.32 | 0.14-0.72 | 0.0063 |
| CD, Crohn’s disease; UC, Ulcerative colitis; IBD, Inflammatory bowel disease; HR, Hazard ratios; CI, Confidence interval | | | | | | |

**Figure S1. Crude mortality rates by era in IBD cases and matched controls.** Crude mortality rates per 100,000 person-years are shown for IBD cases and matched controls, stratified by era (pre-biologic era, 1984–2000; biologic era, 2001–2019). Each panel displays one cause of death. Rates are plotted for cases and controls across eras.


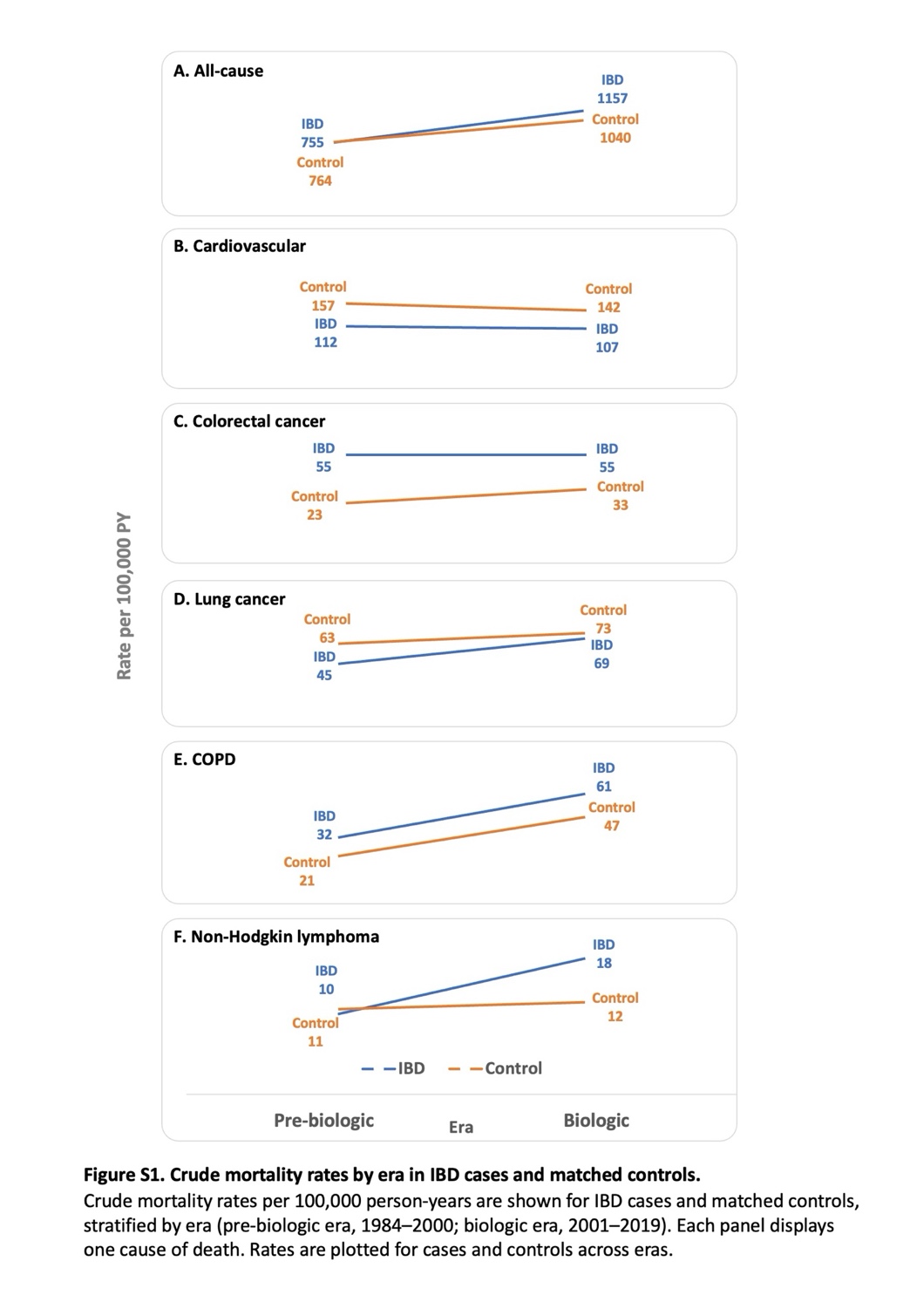

Supplement: jjag105_Supplementary_Data [file jjag105_supplementary_data.zip]
